# A Single‐Cell‐Derived Proliferative Signature Is Associated With Progression‐Free Survival During Immunotherapy and Sunitinib Treatment in Clear Cell Renal Cell Carcinoma

**DOI:** 10.1155/genr/4050985

**Published:** 2026-11-09

**Authors:** Zeyu Li, Shaoqiang Xing, Sheng Zeng, Yifei Zhang, Yaowen Li, Qian Liu

**Affiliations:** ^1^ Department of Urology Tianjin First Central Hospital Tianjin 300071 China tj-fch.com; ^2^ The First Central Clinical School Tianjin Medical University Tianjin 300071 China tijmu.edu.cn; ^3^ Department of Urology The First Affiliated Hospital of Xinxiang Medical University Weihui Henan 453100 China xxmu.edu.cn; ^4^ Tianjin First Central Hospital Nankai University Tianjin 300071 China nankai.edu.cn

**Keywords:** clear cell renal cell carcinoma, immune checkpoint inhibition, proliferation, single-cell RNA sequencing, transcriptomic signature, VEGFR-targeted therapy

## Abstract

**Background:**

Resistance to immune checkpoint inhibitors and VEGFR‐targeted agents limits durable disease control in clear cell renal cell carcinoma (ccRCC). We examined a tumour cell proliferative transcriptional programme in relation to treatment response and progression‐free survival (PFS).

**Methods:**

We analysed predominantly unpaired baseline and progression single‐cell transcriptomes from anti‐PD‐1‐treated ccRCC. A fixed 20‐gene signature underwent patient‐adjusted pseudobulk and RNA‐inferred copy‐number analyses. Pretreatment scores were evaluated in 332 patients across four immunotherapy or sunitinib groups from three studies, with established proliferation benchmarks and time‐varying survival analyses.

**Results:**

The discovery comparison included five baseline and six progression patients. All 20 genes, including PLK1, UBE2C and CDC20, retained higher expression in the proliferative cluster after patient adjustment, although its abundance difference did not survive multiple‐testing correction (BH‐adjusted *q* = 0.164). The score closely tracked G2M (*r* = 0.915–0.980), without superior PFS discrimination in the primary benchmark. Inclusive response AUCs were 0.519 for IMmotion150 immunotherapy, 0.569 for IMmotion150 sunitinib and 0.693 for E‐MTAB‐3267. In E‐MTAB‐3267, the age‐ and sex‐adjusted HR was 1.54 per score standard deviation (95% CI 1.13–2.11). The IMmotion150 immunotherapy association weakened over follow‐up; excluding this nonproportional estimate yielded a pooled HR of 1.51 (95% CI 1.24–1.83) in 158 patients.

**Conclusion:**

Single‐cell and bulk transcriptomic analyses connect a proliferative gene‐expression programme with adverse PFS during immunotherapy and sunitinib treatment in ccRCC. The signature provides a compact transcriptional readout of proliferation associated with treatment outcomes, with a stronger relationship to PFS than to objective response.

## 1. Introduction

Clear cell renal cell carcinoma (ccRCC) is the predominant histological form of renal cell carcinoma and accounts for most kidney cancer deaths [1]. Immune checkpoint inhibitors (ICIs) and vascular endothelial growth factor receptor (VEGFR)–directed tyrosine kinase inhibitors (TKIs) have changed the treatment of advanced disease. For example, pembrolizumab plus axitinib improved survival and objective response compared with sunitinib in a randomised phase 3 trial [2]. Nevertheless, treatment responses vary, and durable disease control remains difficult to predict from pretreatment tumour profiles.

Transcriptomic studies have linked this variation to several interacting biological programmes. IMmotion150 associated angiogenesis, T‐effector/interferon‐γ signalling and myeloid inflammation with outcomes during atezolizumab‐containing therapy and sunitinib [3]. Analysis of IMmotion151 identified molecular subsets distinguished by angiogenic, immune, stromal, metabolic and cell‐cycle activity [4]. Separately, Beuselinck and colleagues described ccRCC subtypes associated with sunitinib response and survival [5]. These studies indicate that a tumour’s proliferative activity should be considered alongside its vascular and immune characteristics, rather than assumed to identify resistance to one treatment.

Single‐cell sequencing can locate these transcriptional programmes within individual cell populations. Bi and colleagues identified cancer‐cell states differing in angiogenic signalling and immune‐evasion programmes in advanced RCC [6], while Krishna and colleagues related multiregional immune states to treatment efficacy [7]. The HCRN study connected tertiary lymphoid structures and exhausted tissue‐resident T cells with response to PD‐1 blockade and made baseline and progression single‐cell profiles available for further analysis [8]. Proliferation itself is not a new prognostic concept: a cell‐cycle progression score has been associated with subsequent metastasis after resection of localised ccRCC [9]. What remains useful to establish is how a compact programme defined in tumour cells relates to outcomes in treated advanced disease and whether it adds information beyond established proliferation measures.

We characterised a proliferative cluster in the HCRN dataset, retained a fixed 20‐gene set from the original cluster screen and evaluated its rank‐based score in four treatment groups from three clinical studies. Patient‐adjusted expression analysis and RNA‐inferred copy‐number profiles were used to examine the single‐cell evidence. We then compared the score with G2M and E2F signatures and assessed objective response, PFS and variation in the PFS association over follow‐up.

## 2. Materials and Methods

### 2.1. Study Design and Cohort Roles

This retrospective study integrated a predominantly unpaired single‐cell discovery series with four pretreatment transcriptomic analysis groups from three clinical studies. The HCRN single‐cell dataset was used to characterise a candidate tumour cell population with higher observed abundance in samples collected at anti‐PD‐1 progression and to define its genes. The resulting gene set was then frozen before analysis of response or progression‐free survival (PFS) in the clinical cohorts. IMmotion150 atezolizumab‐containing arms and CheckMate 009 formed the ICI evaluation series, whereas the IMmotion150 sunitinib arm and E‐MTAB‐3267 formed the VEGFR‐directed TKI series. No signature coefficients or gene weights were estimated, and no outcome‐optimised score cut‐offs were selected in these cohorts.

### 2.2. Data Acquisition and Sample Eligibility

The discovery data were downloaded from Dryad (doi:10.5061/dryad.dv41ns28r) and accompany the HCRN study of anti‐PD‐1‐treated renal cell carcinoma [8]. The archive contained 10*x* Genomics‐filtered feature‐barcode matrices and clinical annotations for 19 samples from 17 patients. We restricted the analysis to 14 samples from 13 patients annotated as ccRCC. One patient contributed both a baseline and a progression sample; to retain one primary discovery observation per patient, the baseline sample was excluded and the progression sample was retained. Specimens with at least 50 candidate tumour cells entered the abundance analysis. The technical eligibility requirement of at least five eligible samples per time point was met by five baseline and six progression samples. After quality control, the atlas comprised 62,344 cells; 7188 candidate tumour cells from the 11 eligible patients entered the primary comparison.

Pretreatment expression and clinical data were assembled for IMmotion150 [3], CheckMate 009 [10] and E‐MTAB‐3267 [5]. The first two datasets were obtained as iAtlas‐harmonised cBioPortal resources [11, 12], under study identifiers rcc_iatlas_immotion150_2018 and ccrcc_iatlas_choueiri_2016, respectively. IMmotion150 was separated into atezolizumab monotherapy, atezolizumab plus bevacizumab and sunitinib groups according to the trial annotation. E‐MTAB‐3267 was obtained from ArrayExpress/BioStudies and processed from raw Affymetrix Human Gene 1.0 ST CEL files. Cohort membership, treatment, available outcomes and analysis sample sizes were reconciled before signature scoring.

### 2.3. Single‐Cell Quality Control and Integration

Filtered feature‐barcode matrices were read with Read10X in Seurat 5.4.0 and SeuratObject 5.3.0, and a Seurat object was created for each sample with a minimum of three cells per gene and 200 detected genes per cell [13]. Cells were retained when they contained 200–7500 detected genes, at least 500 unique molecular identifiers and no more than 25% mitochondrial transcripts. Assay layers were joined after merging the sample‐level objects. Counts were log‐normalised with NormalizeData; 2500 highly variable genes were selected with FindVariableFeatures, scaled and used for principal component analysis with 30 components.

Sample‐associated technical variation was addressed with Harmony 2.0.2 using sample identity as the grouping variable [14]. The all‐cell shared‐nearest‐neighbour graph used Harmony dimensions 1–25. Louvain clustering was performed at a resolution of 0.35, and the same 25 dimensions were used for uniform manifold approximation and projection (UMAP) [15]. A fixed random seed of 20,260,812 was used for stochastic single‐cell steps.

### 2.4. Lineage Annotation and RNA‐Inferred Copy‐Number Assessment

Lineage scores were calculated as the mean log‐normalised expression of genes present from four marker panels. The candidate tumour panel comprised CA9, NDUFA4L2, EPCAM, KRT8, KRT18, KRT19, NNMT, VEGFA and CP; the immune panel comprised PTPRC, LST1, TYROBP, FCER1G, CD3D, CD3E, NKG7, CD79A and MS4A1; the endothelial panel comprised PECAM1, VWF, KDR, EMCN, ENG and RAMP2; and the fibroblast panel comprised COL1A1, COL1A2, COL3A1, DCN, COL6A1 and COL6A2. Cell‐level scores were summarised by their median within each all‐cell cluster.

A cluster was assigned to the putative malignant compartment when its candidate tumour score was positive, exceeded the largest immune, endothelial or fibroblast score by more than 0.5, and its immune score did not exceed 0.25. Cells with detectable PTPRC counts were excluded from malignant‐cell analyses, including PTPRC‐positive cells within otherwise malignant clusters. Candidate cells were reprocessed using 2000 variable genes, 30 principal components, Harmony integration by sample, a neighbour graph based on Harmony dimensions 1–20, clustering at resolution 0.45 and UMAP based on the same dimensions. Copy‐number support was subsequently assessed in the 11 primary‐analysis samples with inferCNV 1.26.0 (Trinity CTAT Project; https://github.com/broadinstitute/inferCNV). Original counts were matched to GENCODE v48 basic autosomal gene coordinates. For each sample, immune references were drawn from its annotated immune cells with detectable PTPRC and at least three combined counts across CD3D, CD3E, NKG7, GNLY, LST1, FCER1G, MS4A1 and CD79A. Up to 300 cells were used as references, and up to 100 separate immune cells were held out for calibration, with minimum numbers of 50 and 25, respectively. InferCNV used a mean‐expression cut‐off of 0.1, a 101‐gene smoothing window, at least three cells per gene, denoising with an SD multiplier of 1.5 and no hidden Markov model. A candidate cell received RNA‐inferred CNV support when its mean squared deviation from neutral expression exceeded the 99th percentile of the held‐out immune distribution in the same sample. Cells below this threshold retained uncertain status. For plotting, denoised profiles were averaged within 5 Mb genomic bins and within each sample–cell‐group combination. Marker counts were normalised to each cell’s original total RNA count, multiplied by 10,000 and log1p‐transformed; the mean‐expression and expressing fraction were first computed per sample. A marker absent from a sample’s filtered input was recorded as missing, not as zero.

### 2.5. Patient‐Level Cluster‐Abundance Screen

For each eligible sample, the abundance of a candidate tumour cluster was its cell count divided by the total number of candidate tumour cells in that sample. Baseline and progression abundances were compared using a one‐sided Wilcoxon rank‐sum test in the adverse direction. Benjamini–Hochberg correction was applied across all 13 screened clusters. Two‐sided tests with the same correction were evaluated as a sensitivity analysis. A cluster was eligible for prioritisation when it was represented in at least four patients, occurred in at least three progression samples, received less than 50% of its cells from any single patient, showed a positive median progression‐minus‐baseline difference, retained a positive difference in at least 80% of leave‐one‐patient‐out analyses and had a one‐sided screening *P* value below 0.10. Among clusters meeting these criteria, the one with the largest median difference was carried forward. The original prioritisation rule is reported to make signature selection reproducible; the revised abundance inference uses the full 13‐test correction. The sole paired patient was summarised separately without a paired hypothesis test.

### 2.6. Fixed Signature and Patient‐Adjusted Expression Analysis

Positive markers of the selected cluster were identified against all other candidate cells using Seurat’s Wilcoxon test (FindMarkers, only.pos = TRUE, minimum detection fraction 0.10, log2 fold change threshold 0.15). Candidate genes had to be detected in at least 40% of selected‐cluster cells and no more than 30% of other candidate cells. Candidates were ordered by average log2 fold change, and the leading 20 genes were frozen as the state definition: CENPA, NUF2, PLK1, UBE2C, CDC20, CDCA3, HMMR, TOP2A, CKAP2L, GTSE1, KIF4A, KIFC1, ASPM, AURKB, KIF14, CCNA2, CCNB2, KIF23, BIRC5 and CDCA8. Patient‐level marker support was assessed by summing original UMI counts within each patient and cell state (cluster 4 or other candidate cells), thereby retaining patients as the biological replicates [16]. Patients contributed both states with at least 20 cells per state in the main analysis; a fixed sensitivity analysis required at least five cells per state. Pseudobulk counts were analysed with edgeR 4.8.2 [17, 18] using filterByExpr, TMM normalisation [19], robust dispersion estimation and a quasilikelihood model with patient and state terms. Benjamini–Hochberg correction covered all genes retained by expression filtering. The original 20 genes were examined without reselection, and the models were repeated after excluding each eligible patient in turn. A sensitivity analysis restricted the counts to CNV‐supported cells while retaining the original cluster labels and 20 genes. The same cell count thresholds were applied; pseudobulk models required at least three patients with both states. The restricted abundance comparison retained the original requirements of at least 50 candidate cells per sample and five samples per sampling group.

### 2.7. Functional Enrichment and Single‐Cell State Summaries

Functional enrichment used the complete expression‐filtered patient‐adjusted pseudobulk result, including positive and negative effects. Genes were ranked by the sign of log2 fold change multiplied by the square root of the quasilikelihood F‐statistic. Numerically negative F values close to zero with *p* = 1 were assigned a rank of zero without removing their genes. All 50 human Hallmark sets were retrieved from the official MSigDB gene‐symbol JSON resource on 8 September 2026 [20]. Gene set enrichment analysis [21] was performed with fgsea 1.36.2 (Korotkevich and colleagues; https://bioconductor.org/packages/fgsea), using a standard two‐sided enrichment statistic, represented set sizes of 15–500 genes, eps = 0 and 10,000 simple permutations. Benjamini–Hochberg correction covered all 50 pathways. The 20‐cell‐per‐state analysis was primary, and the five‐cell analysis was repeated as a sensitivity analysis.

The single‐cell state score was the mean log‐normalised expression of the 20 fixed genes. Sample–cluster means were displayed for combinations containing at least 10 cells. These summaries used the original cluster assignments and did not treat individual cells as independent replicates.

### 2.8. Bulk Transcriptomic Preprocessing

For the iAtlas‐harmonised expression matrices, rows sharing a gene symbol were averaged before scoring. The IMmotion150 matrix contained TPM values; the CheckMate 009 profile was supplied as log2 upper‐quartile‐normalised counts. These processed matrices were used directly for within‐sample ranking. E‐MTAB‐3267 arrays were read with oligo 1.72.0 [22] and Biobase 2.68.0 within Bioconductor [23], then background corrected, quantile normalised and summarised by robust multi‐array average at the transcript‐cluster level [24]. Transcript clusters were mapped from probe identifiers to gene symbols with AnnotationDbi 1.70.0 and hugene10sttranscriptcluster.db 8.8.0. When more than one probe mapped to a gene, the probe with the largest interquartile range across samples was retained; probe identifier resolved any remaining tie. All 20 signature genes were represented in every clinical cohort.

### 2.9. Rank‐Based Signature Scoring

To apply a common scoring rule to the available processed expression matrices, all measured genes were ranked within each sample with average ranks for ties. Each rank was divided by the number of measured genes, and the state score was the mean percentile rank of the 20 frozen genes. Thus, a higher value indicated coordinated placement of the signature genes near the upper end of that sample’s transcriptome. Scores were standardised to mean zero and standard deviation (SD) one within each cohort for Cox modelling. The unstandardised percentile‐rank score was retained for response plots and discrimination analyses. HALLMARK_G2M_CHECKPOINT was the primary proliferation comparator and HALLMARK_E2F_TARGETS the secondary comparator. Their official MSigDB human gene‐symbol JSON files were downloaded on 8 September 2026, with the source files and gene coverage retained. Comparator scores used the same within‐sample ranking calculation. Pearson’s and Spearman’s correlations were assessed in identical patient sets. A sensitivity analysis removed genes overlapping the frozen signature from each comparator, leaving the 20‐gene score unchanged. Absolute Pearson’s correlation of at least 0.85 or a two‐score variance inflation factor above five flagged substantial collinearity. The primary performance comparison used the same IMmotion150 ICI patients at six months, estimating the paired difference in IPCW AUC between the 20‐gene score and G2M. E2F comparisons, the remaining cohorts and the 12‐month horizon were secondary. Collinear score pairs were not entered together into a Cox model. Technical stability was assessed by omitting each gene in turn and by drawing 200 random subsets with 1, 3, 5 or 10 genes removed per clinical group. Scores were recalculated as the mean of the retained genes’ original within‐sample ranks. We summarised correlation, mean absolute difference and maximum absolute difference across patients from the full score; simulation intervals were the 2.5th and 97.5th percentiles across subsets.

### 2.10. Clinical Outcomes and Analysis Populations

PFS was defined from treatment initiation to progression or death using the status and follow‐up supplied with each study. The IMmotion150 ICI series included 174 patients and 109 events; models were adjusted for atezolizumab monotherapy versus atezolizumab plus bevacizumab. CheckMate 009 included 16 nivolumab‐treated patients and 13 events. The IMmotion150 sunitinib group included 89 patients and 55 events, and E‐MTAB‐3267 included 53 patients and 39 events. Additional Cox models included age and sex in E‐MTAB‐3267 and age alone in a small‐sample sensitivity analysis of CheckMate 009. Covariates were not selected by univariate significance. Complete cases were used for each model, with score standardisation retained from the full cohort. Scaled Schoenfeld residuals were used to assess proportional hazards. Where the score term violated this assumption, a score‐by‐log (time in months) interaction was fitted and time‐specific HRs were estimated at 3, 6 and 12 months. The interaction was evaluated by a likelihood‐ratio test against the model without the time interaction. The principal revised constant‐effect synthesis excluded the IMmotion150 ICI group and combined the remaining three unadjusted log HRs by inverse‐variance weighting. Fixed‐effect and DerSimonian–Laird estimates were compared [25]. Legacy four‐group summaries were retained only to show the effect of the revised analysis. Within the three‐study, PH‐compatible set, we omitted one source study at a time and performed 1000 patient bootstrap samples, resampling independently within each study and retaining the original full‐cohort score standardisation. For Kaplan–Meier displays, the cohort median was the fixed cutpoint, with equality assigned to the high‐score group; confidence bands were log–log intervals and risk tables counted patients still under observation immediately before each stated time. Median‐split curves were compared using unstratified log‐rank tests (survdiff, rho = 0); these supporting comparisons used unadjusted *p* values.

The main response analysis compared complete or partial response (CR/PR) with stable or progressive disease (SD/PD), using the response categories supplied by the source studies. This distinction between objective response and nonresponse follows the RECIST framework [26]; imaging was not re‐adjudicated. Patients with missing response data were excluded from this analysis. Ten E‐MTAB‐3267 records labelled only as clinical benefit could not be assigned to a RECIST category and were excluded from the response denominator. CheckMate 009 used the source responder classification. Response‐evaluable populations comprised 165 IMmotion150 ICI patients (48 CR/PR and 117 SD/PD), 82 IMmotion150 sunitinib patients (24 CR/PR and 58 SD/PD) and 43 E‐MTAB‐3267 patients (19 PR and 24 SD/PD). The 16 CheckMate 009 patients comprised three source‐classified responders and 13 nonresponders. The former CR/PR‐versus‐PD comparison was retained as an exploratory extreme‐group analysis. AUC used responders as the positive class and the negative score as the marker, with 95% percentile CIs from 2000 outcome‐stratified patient bootstrap samples. E‐MTAB‐3267 was also examined after excluding each of its five PD cases. Response rates included all evaluable SD cases and used exact binomial CIs. PFS discrimination was evaluated at 6 months and, secondarily, 12 months with timeROC 0.4.1 [27] using inverse‐probability‐of‐censoring weights (IPCW) estimated by the marginal Kaplan–Meier method. Patients censored before a horizon contributed to estimation of the censoring distribution. Events occurring on the horizon were included as cases. Higher scores indicated greater event risk. Pointwise 95% CIs and paired AUC differences were based on the iid representation. A horizon required at least five observed cases, five observed controls and an estimated censoring‐survival probability of at least 0.1; estimates failing these requirements were not reported. Arm‐specific IMmotion150 response analyses used the same inclusive definition; arm‐specific response rates retained the pooled ICI median cutpoint.

### 2.11. Statistical Analysis

Initial single‐cell processing used R 4.5.1; revision analyses used R 4.5.2, including survival 3.8–6 and the packages specified above. Patients were the independent units. Tests were two‐sided except for the original one‐sided cluster screen. Benjamini–Hochberg correction covered the 13‐cluster, genome‐wide expression, 50‐pathway, four‐primary‐PFS and 15‐secondary‐AUC families. Estimates are reported with 95% confidence intervals. Missing data were excluded only from the relevant analysis; stochastic procedures used recorded fixed seeds. Adjusted *q* < 0.05, or two‐sided *p* < 0.05 outside these families, indicated statistical significance. OpenAI Codex assisted with analysis‐code development, source cross‐checking and plotting‐code preparation; estimates and figures were computed from the public data using the R and Python workflows.

## 3. Results

### 3.1. Single‐Cell Profiling and RNA‐CNV Analysis Characterise the Candidate Tumour Compartment

The ccRCC atlas contained 62,344 quality‐controlled cells from 14 samples and 13 patients, including 7533 candidate tumour cells and distinct immune, endothelial, fibroblast and mixed populations (Figure 1A). Patient‐level marker summaries distinguished renal epithelial expression from the held‐out immune population (Figure 1B). Eleven independent samples, comprising five baseline and six progression samples, contributed 7188 candidate cells to the primary comparison (Figure 1C). Genome‐ordered RNA‐CNV profiles and group‐level burdens varied across samples (Figure 1D,E). Overall, 3583 candidate cells (49.8%) exceeded their within‐sample held‐out immune threshold, including 293 of 863 cluster 4 cells (34.0%; Figure 1F). After CNV restriction, only two baseline and six progression samples retained at least 50 cells, so the restricted abundance comparison did not meet the five‐per‐group requirement (Figure 2H).

**FIGURE 1:**
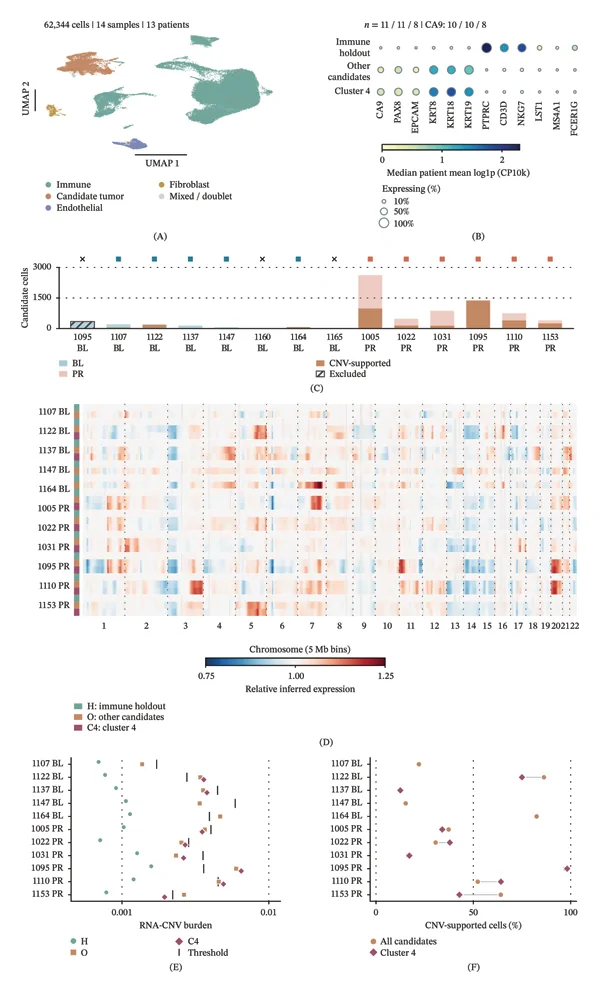
Single‐cell atlas and RNA‐inferred copy‐number assessment. (A) Original whole‐cell UMAP for 62,344 quality‐controlled cells from 14 ccRCC samples and 13 patients, coloured by major lineage. (B) Marker expression in held‐out immune cells, other candidate tumour cells and cluster 4 across the 11 primary samples. Colour shows the median of patient‐level mean log1p‐normalised counts per 10,000 original RNA counts; dot area shows the median expressing fraction. CA9 was unavailable in the filtered input for the immune and other candidate groups of sample 1147 BL and was not assigned zero expression. (C) Candidate‐cell counts, primary eligibility and CNV‐supported counts were assessed. The upper strip uses squares for eligible samples and crosses for exclusions. (D) Mean inferCNV relative expression within sample–cell‐group combinations in 5 Mb autosomal bins. H, held‐out immune; O, other candidates; C4, cluster 4. Missing groups or bins remain unfilled. (E) Median RNA‐CNV burden in each sample and cell group, with the sample‐specific held‐out immune 99th‐percentile threshold. Burden is the mean squared deviation of denoised relative expression from 1, displayed on a logarithmic axis. (F) CNV‐supported fractions among all candidate cells and cluster 4. Totals are 3583/7188 and 293/863, respectively; samples without cluster 4 have no corresponding fraction. RNA‐inferred CNV profiles are relative expression measurements, not direct DNA copy counts. BL, baseline; PR, progression.

**FIGURE 2:**
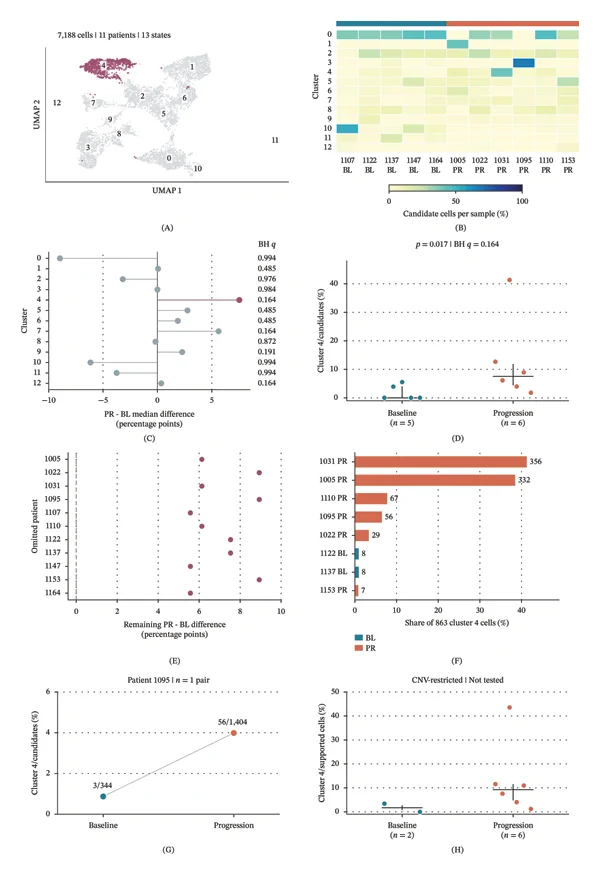
Patient‐level cluster abundance and multiple‐testing correction. (A) Candidate‐cell embedding for the 7188 cells from 11 primary samples, retaining the original clusters and highlighting cluster 4. (B) All 13 cluster proportions by sample, ordered by baseline/progression status and patient identifier. Each denominator is the sample’s total candidate‐cell count; zeros are retained. (C) Progression‐minus‐baseline median abundance differences in percentage points and BH‐adjusted *q* values across all 13 one‐sided tests. (D) Cluster 4 proportions in five baseline and six progression patients. Points are patients; summary marks show medians and interquartile ranges. The one‐sided *p* value is 0.017, and BH q is 0.164. (E) Median differences after omitting each patient in turn. (F) Contributions of eight patients to the 863 cluster 4 cells, with cell counts shown. (G) The single paired patient, 1095: 3/344 candidate cells at baseline and 56/1404 at progression. Only the progression sample is retained in the independent‐patient comparison. (H) Cluster 4 proportions after restriction to CNV‐supported candidates. Only two baseline and six progression samples retain at least 50 cells, below the five‐per‐group requirement; points and median/interquartile summaries are therefore descriptive. BL, baseline; PR, progression.

### 3.2. The Cluster 4 Abundance Difference Does Not Remain Significant After Multiple‐Testing Correction

Cluster 4 was localised within the original candidate‐cell embedding (Figure 2A). The 13‐cluster screen showed substantial between‐patient variation, and no cluster met the BH‐adjusted *q* < 0.05 threshold (Figure 2B,C). Cluster 4 had median abundances of 0% at baseline and 7.53% at progression (one‐sided *p* = 0.017; BH *q* = 0.164; Figure 2D). The two‐sided analysis gave *p* = 0.034 and BH *q* = 0.148. Its positive median difference was retained in all 11 leave‐one‐patient‐out comparisons (Figure 2E). Thus, the observed direction was stable to single‐patient omission, although the corrected screen did not establish a progression‐associated abundance change.

Cluster 4 comprised 863 cells from eight patients; the largest sample contribution was 356 cells (41.25%; Figure 2F). The only patient with both sampling times had 3 of 344 candidate cells in cluster 4 at baseline and 56 of 1404 at progression (Figure 2G). This pair was kept separate from the independent‐patient comparison and was not used to estimate longitudinal expansion.

### 3.3. Patient‐Adjusted Expression Analyses Support the Fixed 20‐Gene Programme

Five progression patients contributed both states at the primary threshold of 20 cells per state, yielding ten pseudobulk libraries and 15,199 tested genes (Figure 3A,B). All 20 genes from the original signature had higher expression in cluster 4, with patient‐adjusted log_2_ fold changes of 4.71–6.39 and a maximum genome‐wide BH *q* of 0.00174. The five‐cell sensitivity analysis included eight patients, comprising two baseline and six progression patients, and retained positive effects for all 20 genes (Figure 3C). Individual‐patient contrasts showed that the programme was distributed across patients, although expression differences were smaller for some genes in the baseline samples (Figure 3D).

**FIGURE 3:**
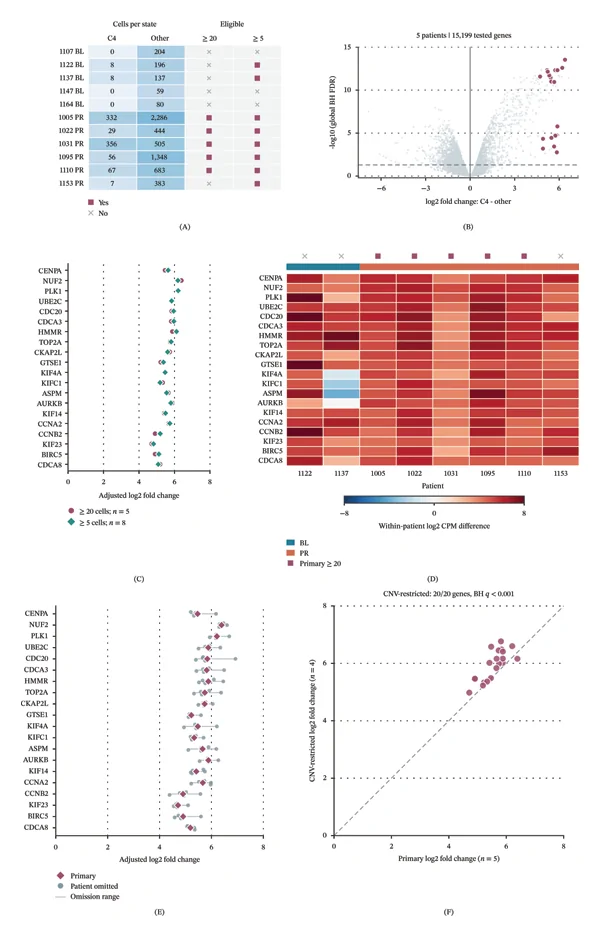
Patient‐adjusted expression of the fixed 20‐gene programme. (A) Cell counts and eligibility for the within‐patient cluster 4 versus other candidate pseudobulk comparison. Five progression patients meet the primary threshold of at least 20 cells per state; two baseline and six progression patients meet the five‐cell sensitivity threshold. (B) Patient‐blocked edgeR quasilikelihood results for all 15,199 tested genes in the primary analysis. The fixed 20 genes are highlighted, and the horizontal reference denotes genome‐wide BH *q* = 0.05. (C) Log_2_ fold changes for the fixed genes under the 20‐cell and five‐cell thresholds, involving five and eight patients, respectively. (D) Within‐patient Log_2_ CPM differences for all 20 genes in the eight‐patient sensitivity set. Aligned strips identify sampling time and membership in the primary model; squares identify primary‐model patients, crosses identify sensitivity‐only patients, and the colour scale retains negative values. (E) Primary effects and leave‐one‐patient‐out effects. Small points show individual omission estimates, and thin ranges span those estimates rather than confidence intervals. (F) Primary effects versus effects after restriction to CNV‐supported cells, leaving four eligible patients at the 20‐cell threshold. The dashed line denotes equal effects; all 20 genes have genome‐wide BH *q* < 0.001 in the CNV‐restricted four‐patient model. The gene list and cluster assignments are fixed throughout. BL, baseline; PR, progression.

All 100 gene‐by‐patient‐exclusion contrasts remained positive in the primary leave‐one‐patient‐out analysis (Figure 3E); the eight‐patient sensitivity analysis likewise retained positive effects in all 160 contrasts. Restricting the primary model to CNV‐supported cells left four patients. All 20 genes remained higher in cluster 4, with log_2_ fold changes of 4.98–6.77 and genome‐wide *q* < 0.001 (Figure 3F). The six‐patient, five‐cell CNV sensitivity analysis supported the same direction.

### 3.4. The Signature Closely Tracks Established Proliferation Scores

The fixed gene set overlapped G2M checkpoint and E2F target programmes (Figure 4A). Across the four clinical groups, its Pearson’s correlation was 0.915–0.980 with G2M and 0.906–0.969 with E2F (Figure 4B–F). Removing overlapping genes from the comparator sets retained strong correlations of 0.874–0.970 (Figure 4F). All score pairs met the predefined collinearity criterion, indicating that their agreement extended beyond shared gene membership.

**FIGURE 4:**
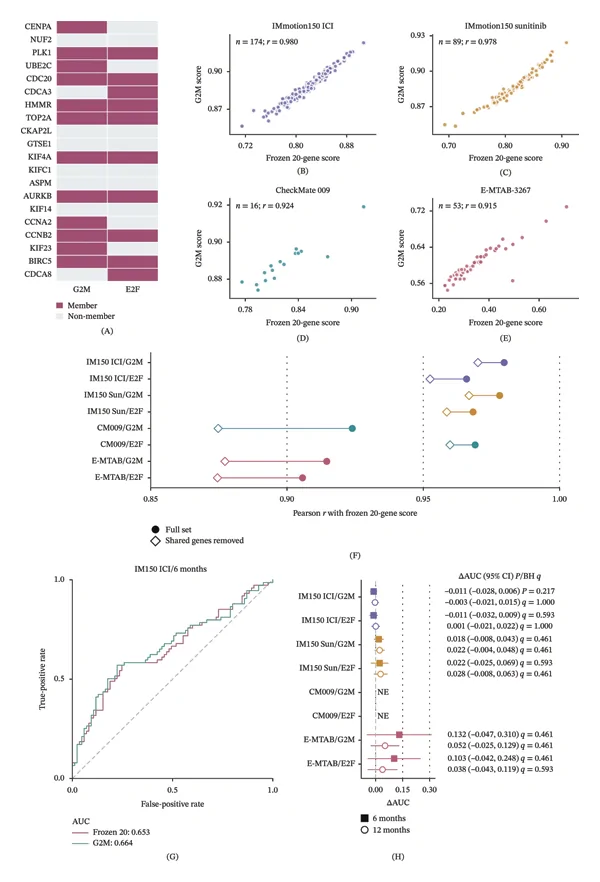
Comparison with established proliferation signatures. (A) Membership of the 20 fixed genes in Hallmark G2M checkpoint and E2F target sets. (B–E) Patient‐level fixed and G2M scores in IMmotion150 ICI (*n* = 174), IMmotion150 sunitinib (*n* = 89), CheckMate 009 (*n* = 16) and E‐MTAB‐3267 (*n* = 53), respectively, with Pearson’s correlations. Axes use the observed score ranges in each group. (F) Pearson’s correlations with the full comparator sets and with sets after removing genes shared with the fixed score. Connected symbols identify the two versions of each comparator in the same patients. (G) Same‐patient IPCW ROC curves for 6‐month PFS in all 174 IMmotion150 ICI patients. (H) Paired differences in six‐ and 12‐month IPCW AUC, defined as the fixed score minus G2M or E2F, with pointwise 95% confidence intervals. The 6‐month IMmotion150 ICI comparison with G2M is primary; other comparisons are secondary. Panel H reports the two‐sided *p* value for the primary comparison and BH‐adjusted q values for the 15 secondary comparisons. NE denotes nonestimability in CheckMate 009 because only three observed controls are available at either horizon. Higher scores predict PFS events. IPCW, inverse‐probability‐of‐censoring weighting. IM150 ICI, IMmotion150 ICI; IM150 Sun, IMmotion150 sunitinib; CM009, CheckMate 009; E‐MTAB, E‐MTAB‐3267.

In the primary comparison using the same 174 IMmotion150 ICI patients, 6‐month IPCW PFS AUC was 0.653 for the 20‐gene score and 0.664 for G2M (Figure 4G). The paired difference was −0.011 (95% CI −0.028 to 0.006; *p* = 0.217). Secondary six‐ and 12‐month comparisons with G2M and E2F were also imprecise, and none established an advantage for the compact score (Figure 4H). CheckMate 009 had only three observed controls at each fixed horizon and did not meet the minimum requirement for time‐dependent AUC estimation.

### 3.5. Complete Transcriptome Rankings Identify a Predominantly Proliferative State

Analysis of all 50 Hallmark pathways placed E2F, G2M and mitotic‐spindle activity among the strongest positive enrichments in the complete patient‐adjusted ranking (Figure 5A). E2F targets had an NES of 3.84 (BH *q* = 1.10 × 10^−83^), G2M checkpoint an NES of 3.75 (*q* = 5.17 × 10^−72^) and mitotic spindle an NES of 3.04 (*q* = 5.17 × 10^−28^; Figure 5B–D). The primary ranking included all 15,199 expression‐filtered genes; the five‐cell sensitivity ranking contained 14,056 genes and retained strong proliferation‐pathway enrichment. Epithelial–mesenchymal transition did not meet the adjusted threshold in the primary analysis (NES = 1.31; *q* = 0.080).

**FIGURE 5:**
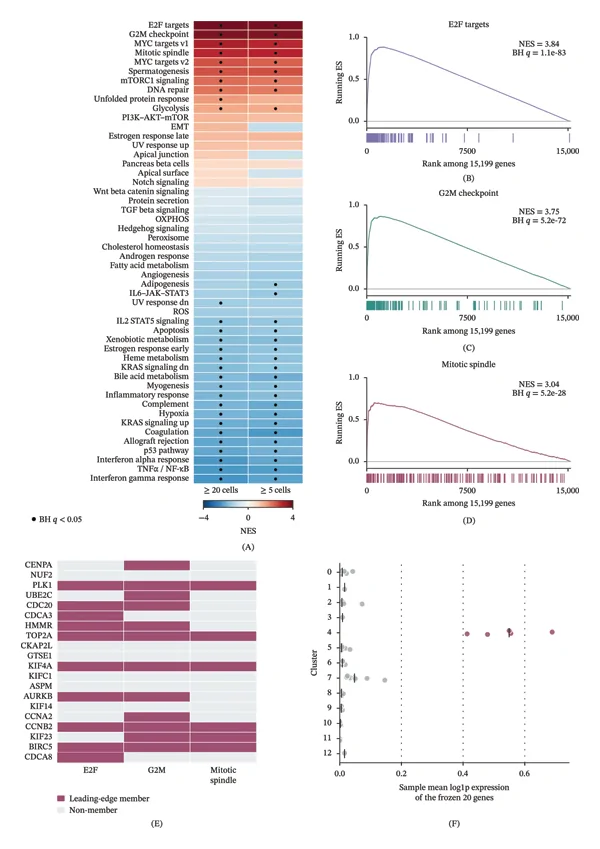
Full‐rank functional enrichment and cellular localisation. (A) Normalised enrichment scores for all 50 Hallmark pathways from complete patient‐adjusted gene rankings at the 20‐cell and five‐cell thresholds, comprising five patients/15,199 genes and eight patients/14,056 genes, respectively. Pathways are ordered by primary NES; dots denote BH *q* < 0.05 within each 50‐pathway family. (B–D) Full‐rank running enrichment curves for E2F targets, G2M checkpoint and mitotic spindle. Rugs locate pathway genes among all 15,199 ranked genes in the primary analysis. (E) Membership of the 20 fixed genes in the three pathway leading edges. (F) Mean log1p expression of the fixed genes across the 13 candidate‐cell clusters. Each point represents one sample–cluster group with at least ten cells; short marks show cluster‐wise medians. There are 62 eligible groups from 11 independent patients, including five eligible samples for cluster 4. Absent or ineligible groups are not assigned zero expression. NES, normalised enrichment score; ES, enrichment score; EMT, epithelial–mesenchymal transition; OXPHOS, oxidative phosphorylation; ROS, reactive oxygen species. Short pathway labels correspond to TNFα signalling via NF‐κB, PI3K–AKT–mTOR signalling and IL6–JAK–STAT3 signalling.

The fixed genes contributed to the leading edges of these proliferative pathways (Figure 5E). Patient‐level expression summaries also localised the 20‐gene programme to cluster 4 across the original cluster assignments (Figure 5F). These results support a coherent cell‐cycle programme rather than a distinct nonproliferative resistance mechanism.

### 3.6. The Score is Technically Stable to Loss of Individual Genes

All 20 genes were represented in each clinical group, and recalculation from the archived expression matrices reproduced all 332 original scores to numerical precision. Median within‐patient gene ranks varied by cohort and platform (Figure 6A). Omitting each gene in turn produced correlations of 0.9966–0.9999 with the complete score across the 80 cohort‐by‐gene comparisons (Figure 6B). The corresponding mean absolute changes quantify shifts in score level separately from correlation (Figure 6C).

**FIGURE 6:**
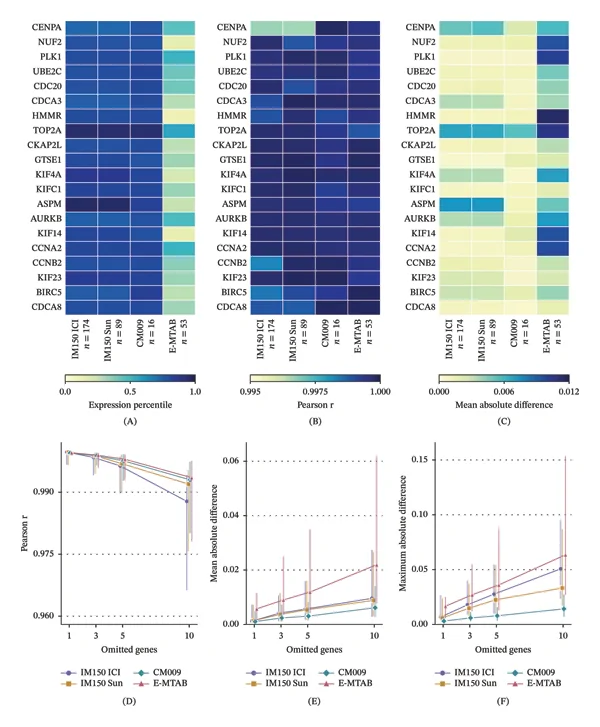
Technical stability of the fixed rank‐based score. (A) Median within‐patient expression percentiles for the 20 fixed genes in IMmotion150 ICI (*n* = 174), IMmotion150 sunitinib (*n* = 89), CheckMate 009 (*n* = 16) and E‐MTAB‐3267 (*n* = 53). All genes are represented in each group. (B, C) Pearson’s correlation and mean absolute score difference after omitting the gene named in each row. Panel B displays raw Pearson’s r on a 0.995–1.000 scale; panel C uses a shared zero‐based scale. (D–F) Correlation, mean absolute score difference and maximum absolute score difference across patients after removing 1, 3, 5 or 10 genes. Points show medians and intervals show the 2.5–97.5th percentiles across 200 random subsets per omission level and clinical group. Within‐sample ranks retain the original expression‐universe denominator; the averaging step uses only retained genes. These are technical gene‐omission intervals, not patient‐sampling confidence intervals. IM150 ICI, IMmotion150 ICI; IM150 Sun, IMmotion150 sunitinib; CM009, CheckMate 009; E‐MTAB, E‐MTAB‐3267.

Across 200 random omissions per level and cohort, median correlations remained 0.988–0.994 when ten genes were removed (Figure 6D). Median mean absolute score differences at that level ranged from 0.0061 to 0.0220, with larger deviations in E‐MTAB‐3267 (Figure 6E). The redundancy among the fixed genes therefore preserved relative patient ordering more strongly than exact score values. Maximum absolute differences show the largest patient‐level change within each omitted‐gene subset (Figure 6F).

### 3.7. Inclusive ICI Response Discrimination is Weaker Than Early PFS Discrimination

The inclusive IMmotion150 ICI response population comprised 165 patients, including 48 CR/PR and 117 SD/PD cases (Figure 7A). Response rates were 22/82 (26.8%) below the pooled median score and 26/83 (31.3%) above it, with no adverse high‐score ordering within either treatment arm (Figure 7B). The inclusive AUC was 0.519 (95% CI 0.427–0.613; two‐sided *p* = 0.702), compared with 0.641 (95% CI 0.533–0.748) in the exploratory 100‐patient CR/PR‐versus‐PD comparison (Figure 7C). Inclusive arm‐specific AUCs were 0.541 for atezolizumab monotherapy and 0.504 for atezolizumab plus bevacizumab, both with intervals spanning 0.5 (Figure 7D).

**FIGURE 7:**
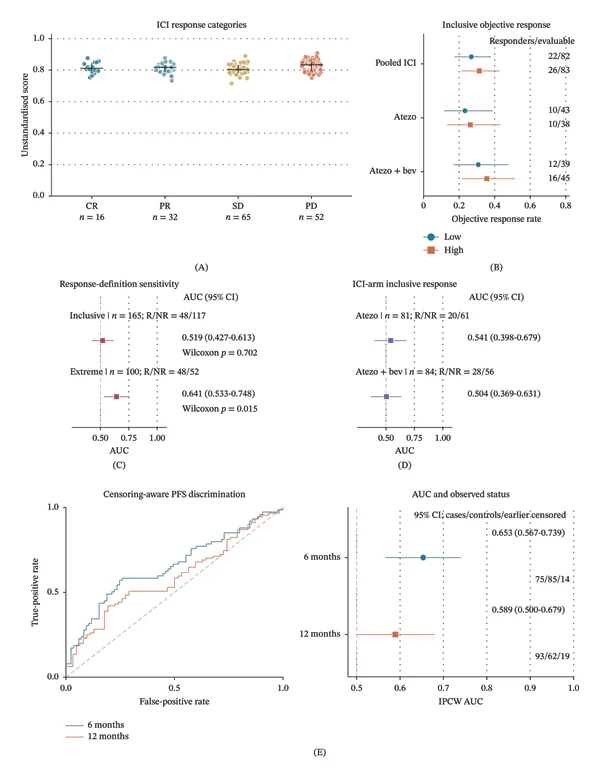
Inclusive ICI response and censoring‐aware PFS discrimination. (A) Unstandardised percentile‐rank scores across CR, PR, SD and PD in 165 response‐evaluable IMmotion150 ICI patients. Points are patients; summary marks show medians and interquartile ranges. (B) Inclusive CR/PR rates overall and within each treatment arm, with exact binomial 95% intervals and responders/evaluable denominators. Low and high groups use the median of all 174 pooled ICI patients, with equality assigned to the high group. (C) Response AUCs for inclusive CR/PR versus SD/PD (*n* = 165; 48/117) and exploratory CR/PR versus PD (*n* = 100; 48/52). The *P* values in C are two‐sided Wilcoxon tests of scores between the response groups in each row. R/NR denotes responder/nonresponder counts. (D) Inclusive arm‐specific AUCs: monotherapy, *n* = 81 (20 responders/61 nonresponders); combination, *n* = 84 (28/56). Response is the positive class, and the marker is the negative score; C and D show 2000 outcome‐stratified bootstrap percentile 95% intervals. (E) Six‐ and 12‐month IPCW PFS ROC curves and AUCs with iid‐based pointwise 95% intervals in all 174 patients. Cases/controls/earlier censorings are 75/85/14 at six months and 93/62/19 at 12 months. Earlier‐censored patients contribute to estimation of the censoring distribution; higher scores predict PFS events. Atezo, atezolizumab; bev, bevacizumab.

PFS discrimination was greater at 6 months than at 12 months. In all 174 ICI patients, IPCW AUC was 0.653 (95% CI 0.567–0.739) at six months and 0.589 (95% CI 0.500–0.679) at 12 months (Figure 7E). Fourteen and 19 patients were censored before the six‐ and 12‐month horizons, respectively; they were not classified as observed cases or controls at those horizons but contributed to estimation of the censoring distribution.

### 3.8. The ICI PFS Association Changes Over Follow‐Up

Median‐split survival curves illustrated earlier PFS events in the high‐score IMmotion150 ICI group (log‐rank *p* = 0.217; Figure 8A). The arm‐adjusted constant‐effect HR was 1.29 per SD (95% CI 1.05–1.58; *p* = 0.0135), but the score‐specific PH test was significant (*p* = 0.0022). The time interaction was supported by the likelihood‐ratio test (*p* = 0.0017). In the arm‐adjusted time‐varying model, the HR was 1.47 at 3 months (95% CI 1.18–1.82), 1.11 at 6 months (95% CI 0.89–1.40) and 0.85 at 12 months (95% CI 0.61–1.19; Figure 8B). The Schoenfeld‐based coefficient diagnostic showed the corresponding decline over follow‐up (Figure 8C).

**FIGURE 8:**
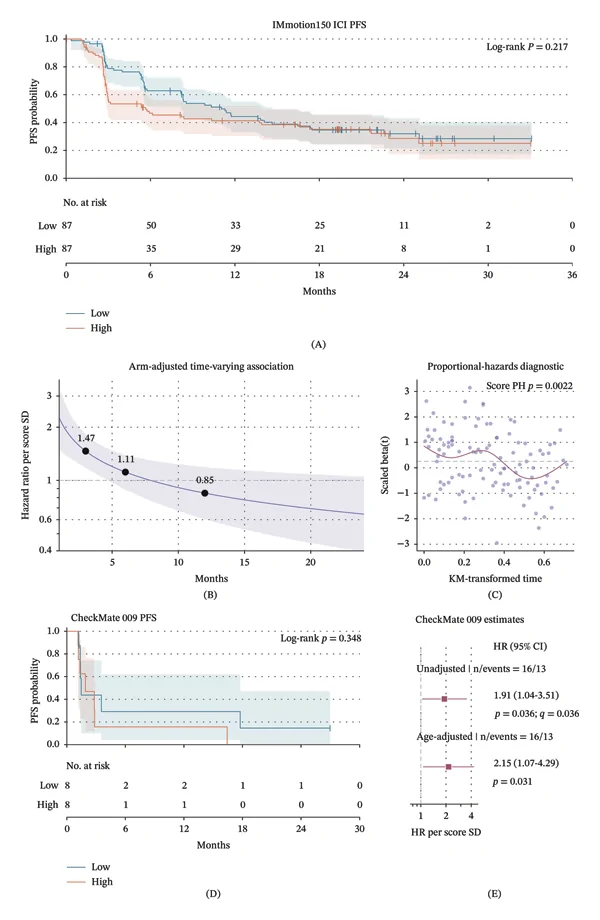
ICI survival and time‐varying score associations (A) IMmotion150 ICI Kaplan–Meier PFS curves by the cohort median score (174 patients; 109 events; log‐rank *p* = 0.217). (B) Arm‐adjusted score‐by‐log (time) model showing the HR per full‐cohort score SD and pointwise 95% band over 1–24 months; markers identify three, six and 12 months. The time interaction likelihood‐ratio test gives *p* = 0.0017. (C) Scaled Schoenfeld‐based coefficient estimates *β* (*t*) at 109 event times against Kaplan–Meier‐transformed time, with a descriptive natural‐spline smooth and constant‐coefficient reference. The score‐specific PH test *p* value is 0.0022. (D) CheckMate 009 median‐split PFS curves (16 patients; 13 events; log‐rank *p* = 0.348). Kaplan–Meier bands are log–log 95% intervals, ticks denote censoring, and risk tables report 6‐month steps over 0–36 months in A and 0–30 months in D. Equality at the median is assigned to the high group. Kaplan–Meier *p* values are from unstratified log‐rank tests and are unadjusted. (E) Unadjusted and age‐adjusted CheckMate 009 HRs per full‐cohort score SD, with 95% intervals. Both models contain 16 patients and 13 events; age adjustment is a small‐sample sensitivity analysis. Forest‐plot *p* values are two‐sided Wald tests of the continuous‐score Cox coefficient; *q* values are BH‐adjusted across the four primary PFS models. n/events denotes patients/PFS events.

CheckMate 009 included 16 nivolumab‐treated patients and 13 PFS events. The median‐split comparison yielded log‐rank *p* = 0.348 (Figure 8D). Continuous‐score estimates showed an adverse association (Figure 8E). The unadjusted HR was 1.91 per SD (95% CI 1.04–3.51; *p* = 0.036), and the age‐adjusted estimate was 2.15 (95% CI 1.07–4.29; *p* = 0.031). The broad intervals reflect the limited precision of this small cohort.

### 3.9. Sunitinib Cohorts Show Adverse PFS Associations and Response‐Definition Sensitivity

Patient‐level response distributions and inclusive response rates are shown in Figure 9A,B. The inclusive response AUC was 0.569 in 82 IMmotion150 sunitinib patients (95% CI 0.434–0.704) and 0.693 in 43 E‐MTAB‐3267 patients (95% CI 0.520–0.844), compared with exploratory extreme‐group AUCs of 0.681 and 0.947 (Figure 9C). Median‐split comparisons yielded log‐rank *p* = 0.190 in IMmotion150 sunitinib and *p* = 0.080 in E‐MTAB‐3267 (Figure 9E,F). Continuous‐score HRs were 1.45 in IMmotion150 sunitinib (89 patients, 55 events; 95% CI 1.10–1.91; *p* = 0.0079) and 1.49 in E‐MTAB‐3267 (53 patients, 39 events; 95% CI 1.10–2.01; *p* = 0.0105). Adjustment for age and sex in E‐MTAB‐3267 yielded HR 1.54 (95% CI 1.13–2.11; *p* = 0.0070), with score‐specific PH *p* = 0.233 (Figure 9G).

**FIGURE 9:**
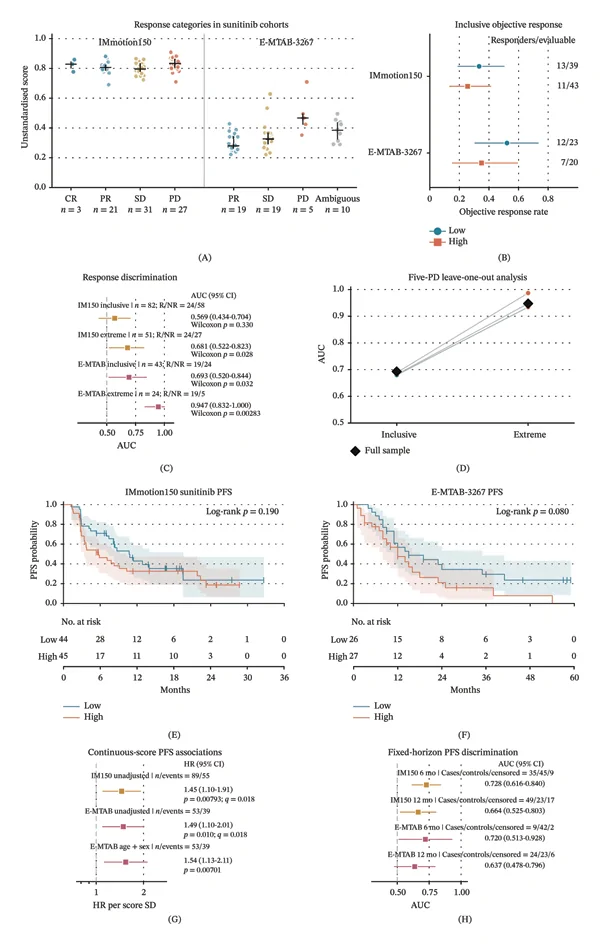
Sunitinib outcomes and response population sensitivity. (A) Unstandardised percentile‐rank scores across response categories in both sunitinib cohorts. Points are patients, with medians and interquartile ranges. Ten E‐MTAB‐3267 clinical‐benefit annotations are shown separately and remain in PFS analyses, but are not assigned to RECIST categories. (B) Inclusive CR/PR rates by cohort median score group, with exact binomial 95% intervals and responders/evaluable denominators; equality is assigned to the high group. Seven missing IMmotion150 responses and ten ambiguous E‐MTAB‐3267 labels are excluded from response calculations. (C) Inclusive and exploratory extreme‐group AUCs, with 2000 outcome‐stratified bootstrap percentile 95% intervals. Inclusive/extreme sample sizes are 82/51 for IMmotion150 sunitinib and 43/24 for E‐MTAB‐3267. Response is the positive class, and the marker is the negative score. The *p* values in C are two‐sided Wilcoxon tests of scores between the response groups in each row; R/NR denotes responder/nonresponder counts. (D) Inclusive and extreme‐group AUCs after omitting each of the five E‐MTAB‐3267 PD patients. Linked points share the omitted patient; each extreme comparison retains 19 PR and four PD cases. Filled diamonds mark full‐sample AUCs. (E, F) Median‐split PFS curves in IMmotion150 sunitinib (89 patients; 55 events) and E‐MTAB‐3267 (53 patients; 39 events), with log–log 95% bands, censoring ticks and risk tables over 0–36 months in 6‐month steps or 0–60 months in 12‐month steps, respectively. The unstratified log‐rank *p* values are 0.190 and 0.080, respectively, without multiplicity adjustment. (G) Unadjusted PFS HRs in both cohorts and the age‐ and sex‐adjusted E‐MTAB‐3267 estimate, per full‐cohort score SD with 95% intervals. (H) Six‐ and 12‐month IPCW PFS AUCs with iid‐based pointwise 95% intervals. Cases/controls/earlier censorings are 35/45/9 and 49/23/17 in IMmotion150 and 9/42/2 and 24/23/6 in E‐MTAB‐3267. Forest‐plot *p* values are two‐sided Wald tests of the continuous‐score Cox coefficient; *q* values are BH‐adjusted across the four primary PFS models. n/events denotes patients/PFS events. IM150, IMmotion150; censored in H denotes censoring before the specified horizon.

Leaving out each of the five E‐MTAB‐3267 PD cases retained the difference between the inclusive and extreme‐group AUCs (Figure 9D). The extreme‐group estimate was based on five PD and 19 PR cases, so its precision remained limited despite stability to individual‐case omission. The response‐category plots also retained ten ambiguous clinical‐benefit annotations as a separate group, without assigning them to CR, PR, SD or PD (Figure 9A). Six‐ and 12‐month IPCW estimates provide the corresponding fixed‐horizon PFS discrimination in both sunitinib cohorts (Figure 9H).

### 3.10. The PH‐Compatible Synthesis Retains an Adverse PFS Association

Excluding the time‐varying IMmotion150 ICI estimate left three independent study groups with 158 patients and 107 PFS events. Their unadjusted inverse‐variance pooled HR was 1.51 per SD (95% CI 1.24–1.83; *p* = 3.16 × 10^−5^; Figure 10B). Score‐specific PH tests in these groups had *p* values above 0.05 (Figure 10A). The estimated between‐study variance was zero, giving identical fixed‐effect and DerSimonian–Laird summaries. The legacy four‐group mixed‐adjustment estimate was 1.40 (95% CI 1.22–1.61), and the uniformly unadjusted four‐group estimate was 1.39 (95% CI 1.21–1.60; Figure 10C).

**FIGURE 10:**
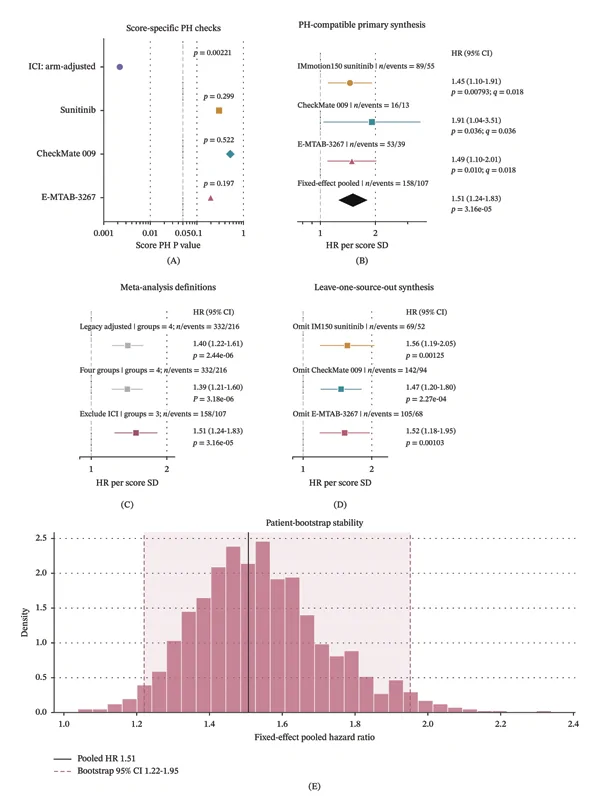
PFS synthesis excluding the nonproportional ICI estimate. (A) Score‐specific proportional hazards tests for the arm‐adjusted IMmotion150 ICI model and unadjusted models in the other three groups. The reference marks *p* = 0.05; patients/events are 174/109, 89/55, 16/13 and 53/39. (B) Unadjusted PFS HRs in IMmotion150 sunitinib, CheckMate 009 and E‐MTAB‐3267, with inverse‐variance fixed‐effect synthesis across 158 patients and 107 events. Effects are per original full‐cohort score SD; intervals are 95% confidence intervals. The diamond denotes the pooled HR. (C) Legacy four‐group mixed‐adjustment, four‐group uniformly unadjusted and revised three‐study summaries. These sensitivity analyses reuse overlapping patients. (D) Leave‐one‐source‐study‐out estimates within the three‐study PH‐compatible set; IMmotion150 ICI remains excluded. Omitting IMmotion150 sunitinib, CheckMate 009 or E‐MTAB‐3267 leaves 69/52, 142/94 or 105/68 patients/events, respectively. (E) Distribution of the pooled HR from 1000 patient bootstrap samples drawn independently within the three studies. Reference lines mark the original estimate and bootstrap percentile limits. The bootstrap median is 1.53, and the percentile 95% interval is 1.22–1.95. Forest‐plot *p* values are two‐sided Wald tests; cohort *q* values are BH‐adjusted across the four primary PFS models. n/events denotes patients/PFS events, and groups denotes contributing treatment‐group estimates rather than independent studies. IM150, IMmotion150.

Within the PH‐compatible set, omitting one source study at a time produced pooled HRs of 1.47–1.56, with all three intervals above 1 (Figure 10D). Resampling patients independently within these studies gave a median pooled HR of 1.53 and a 95% percentile interval of 1.22–1.95 across 1000 replicates (Figure 10E). The wider resampling interval preserved the adverse direction while reflecting uncertainty in the available patient samples.

## 4. Discussion

A proliferative transcriptional programme identified in ccRCC tumour cells was associated with adverse PFS during ICI‐containing therapy and sunitinib treatment. Patient‐adjusted expression analyses supported the 20‐gene programme, and its bulk‐tumour score closely tracked established proliferation measures. The clinical findings were more consistent for PFS than for objective response. Together, these observations link a defined single‐cell population to a compact measure of proliferative activity and place its PFS association within established cell‐cycle biology.

Previous single‐cell studies place this finding within a broader view of ccRCC biology. Bi et al. described cancer‐cell populations with different angiogenic and immunosuppressive programmes and related their signatures to survival [6]. Krishna et al. instead emphasised spatially heterogeneous immune populations, including tissue‐resident T cells and tumour‐associated macrophages associated with therapeutic response [7]. In the HCRN source study, the combination of tertiary lymphoid structures and a lower abundance of exhausted tissue‐resident T cells was linked to response to PD‐1 blockade [8]. Our analysis of that resource focuses on a different compartment: cycling candidate tumour cells. This tumour cell perspective complements the immune context described in the source study.

Convergence on G2M checkpoint, E2F targets and mitotic‐spindle activity gives the signature a clear biological interpretation. Coordinated cell‐cycle transcription can generate prognostic signatures containing different genes but measuring much the same process [28]. Here, correlations with G2M and E2F remained strong even after shared genes were removed, and deleting individual signature genes had little effect on patient ordering. These results favour a common proliferation programme over a separate resistance pathway. The lack of improvement over G2M is informative in this context: the value of the 20‐gene set lies chiefly in tracing that programme to a single‐cell population, rather than in establishing a better clinical predictor.

Proliferation‐associated risk has also been studied in surgically treated kidney cancer. Askeland et al. found that a 31‐gene cell‐cycle progression score was associated with later metastatic progression after resection of apparently localised ccRCC [9]. Rini et al. developed and independently validated a 16‐gene assay for recurrence after nephrectomy in stage I–III disease, with prognostic information beyond clinical and pathological factors [29]. Those studies addressed postoperative recurrence or metastasis, whereas the present analysis concerns PFS after systemic treatment in advanced disease. Differences in the genes, calculations, patient populations and endpoints prevent direct comparison of predictive performance. The biological connection is nevertheless relevant: proliferation‐associated expression can accompany an adverse disease course in both surgical and systemic‐treatment settings.

Treatment context is important when interpreting this association. IMmotion150 linked different immune and angiogenic programmes to outcomes across its treatment arms [3], and the Beuselinck classification associated distinct tumour subtypes with sunitinib response [5]. In IMmotion151, atezolizumab plus bevacizumab showed greater benefit than sunitinib in subsets with high T‐effector and/or cell‐cycle transcription [4]. That relative treatment comparison is not equivalent to the within‐treatment association tested here. An adverse PFS association in both ICI‐ and sunitinib‐treated groups is more consistent with a general proliferation‐associated risk signal than with a marker selecting one therapy over another. Demonstrating differential treatment benefit would require a treatment‐by‐score interaction in an appropriately designed comparative dataset [30].

The timing of progression adds another dimension. In IMmotion150, the score’s PFS association was strongest early and weakened during follow‐up; the six‐ and 12‐month discrimination estimates showed a similar pattern. A single constant HR can obscure such changes when proportional hazards do not hold [31]. We therefore interpret this cohort through the time‐varying estimates and exclude its constant‐effect estimate from the principal pooled analysis. Early progression could reflect a greater growth burden in highly proliferative tumours, although changes in the composition of patients remaining at risk may also contribute. Neither explanation can be distinguished with the available pretreatment profiles. Continuous‐score and median‐split analyses also answer different questions: dividing a continuous measurement at its median loses information [32], but that does not remove the uncertainty shown by the nonsignificant log‐rank comparisons.

Patient‐level replication is particularly important for the single‐cell component. Treating thousands of cells as independent biological replicates can produce misleading differential‐expression evidence [16]. Patient‐blocked pseudobulk models and leave‐one‐patient‐out analyses showed that the selected expression programme was not confined to one patient’s cells, and its direction persisted in the RNA‐CNV‐supported subset. These checks strengthen the characterisation of the programme within the discovery data; they are not independent replication of gene selection. By contrast, the cluster‐abundance difference did not survive multiple‐testing correction, RNA‐inferred CNV support was incomplete, and the primary pseudobulk comparison included progression samples only. The largely unpaired sampling therefore describes between‐patient differences rather than demonstrating treatment‐induced expansion within individual tumours.

A rank‐based calculation allowed the same fixed gene set to be applied across the available RNA‐sequencing and microarray profiles without fitting outcome‐dependent weights. Single‐sample rank scoring has been developed to reduce dependence on the number and composition of background samples [33]. Following this general principle, our custom calculation took the mean percentile rank of the 20 genes. Strong gene‐omission correlations indicate redundancy in relative patient ordering, whereas the observed absolute score differences remain relevant to measurement reproducibility. Gene coverage, preprocessing and platform‐specific score distributions would need to be addressed in any future assay. Establishing a common clinical cut‐off would then require analytical validation and external calibration beyond the cohort medians used here.

Response and PFS should also remain distinct clinical outcomes. RECIST separates objective tumour shrinkage from SD and PD [26]; lack of an objective response does not by itself establish treatment resistance. Including stable disease markedly reduced response discrimination, especially in E‐MTAB‐3267, where the extreme‐group analysis contained only five PD cases. This contrast suggests that the score captures aspects of disease course that a binary shrinkage endpoint does not fully represent. Small validation samples and incomplete IMDC or MSKCC risk information limit how far that interpretation can be taken. Evaluation in contemporary, clinically annotated cohorts should prioritise incremental value over standard risk assessment, while paired tumour sampling could determine whether the proliferative population changes during therapy.

## 5. Conclusion

A fixed 20‐gene signature localised a proliferative programme in ccRCC single‐cell data and was associated with PFS in ICI‐ and sunitinib‐treated cohorts. Strong agreement with established proliferation scores and weak inclusive ICI response discrimination favour its interpretation as a compact proliferation readout associated with adverse outcomes.

## Author Contributions

Zeyu Li: conceptualisation, methodology, software, formal analysis, data curation, visualisation and writing–original draft. Shaoqiang Xing: methodology, validation, formal analysis and writing–review and editing. Sheng Zeng: investigation, data curation and validation. Yifei Zhang: investigation, validation and writing–review and editing. Yaowen Li: data curation, validation and writing–review and editing. Qian Liu: conceptualisation, supervision, project administration and writing–review and editing.

## Funding

This work was supported by the National Natural Science Foundation of China (82472740, 82202323), Tianjin Natural Science Foundation (23JCZDJC00850, 22JCQNJC01640), Tianjin Key Medical Discipline (Specialty) Construction Project (TJYXZDXK‐3‐024C), and Tianjin Health Science and Technology Project (TJWJ2024XK010, TJWJ2024QN042).

## Ethics Statement

This study analysed deidentified data from previously published studies and public repositories. No new participants were recruited, and no new biological samples were collected. Ethics approvals and informed‐consent procedures for collection of the original data are reported in the corresponding source studies.

## Conflicts of Interest

The authors declare no conflicts of interest.

## Data Availability

The public datasets analysed in this study are available from Dryad (HCRN single‐cell data; https://doi.org/10.5061/dryad.dv41ns28r), ArrayExpress/BioStudies (E‐MTAB‐3267) and cBioPortal Datahub (IMmotion150, rcc_iatlas_immotion150_2018; CheckMate 009, ccrcc_iatlas_choueiri_2016). Analysis scripts and processed data supporting the findings are available from the corresponding author upon reasonable request.
